# Multifocal Posterior Pigment Epitheliopathy in a Pachychoroid Eye Following Ahmed Glaucoma Valve Implantation: A Case Report

**DOI:** 10.7759/cureus.116080

**Published:** 2026-09-10

**Authors:** Mayu Katsume, Yuta Kitamura, Yuto Kawamata, Takayuki Baba

**Affiliations:** 1 Department of Ophthalmology and Visual Science, Chiba University Graduate School of Medicine, Chiba, JPN

**Keywords:** exfoliation glaucoma, glaucoma drainage device, intraocular pressure reduction, multifocal posterior pigment epitheliopathy, pachychoroid spectrum

## Abstract

We report a case of multifocal posterior pigment epitheliopathy (MPPE), a bullous variant within the pachychoroid disease spectrum, following Ahmed Glaucoma Valve (AGV) implantation in a patient with exfoliation glaucoma (XFG). An 81-year-old man with XFG underwent AGV implantation for uncontrolled intraocular pressure (IOP) of 48 mmHg despite maximal medical therapy. Preoperative best-corrected visual acuity (BCVA) was 20/20. Preoperative optical coherence tomography (OCT) demonstrated dilated pachyvessels in Haller's layer with attenuation of the inner choroid, consistent with an underlying pachychoroid phenotype, although the subfoveal choroidal thickness was unremarkable at 210 μm. Although postoperative IOP stabilized at 5-9 mmHg without early or hypotony-related structural complications, he developed multifocal pigment epithelial detachments (PEDs) and serous retinal detachment (SRD) in the macula one month postoperatively, accompanied by marked choroidal thickening (subfoveal choroidal thickness increasing from 210 to 362 μm) and pachyvessel dilatation. Fluorescein angiography (FA) revealed multifocal leakage in the superior and inferior nasal macula, and OCT angiography confirmed multiple PEDs overlying dilated choroidal vessels without macular neovascularization, leading to a diagnosis of MPPE. Following focal laser photocoagulation and recovery of IOP to 14 mmHg, the SRD resolved completely by postoperative day 93. However, the final BCVA was 20/320 due to persistent outer retinal changes. This case suggests that acute IOP reduction may have contributed to exudative decompensation by unmasking the latent exudative potential of a pre-existing pachychoroid substrate. Preoperative assessment of choroidal morphology and careful postoperative fundus monitoring should be considered in eyes with pachychoroid features undergoing glaucoma surgery.

## Introduction

Multifocal posterior pigment epitheliopathy (MPPE), first described by Uyama et al., is regarded as a bullous variant of central serous chorioretinopathy (CSC). It is characterized by multiple diffuse exudative lesions in the posterior pole, a flattened exudative retinal detachment in the macular area, and an inferior bullous retinal detachment. Its pathogenesis has been attributed primarily to choroidal vascular hyperpermeability, leading to fluid extravasation and subsequent diffuse dysfunction of the retinal pigment epithelium (RPE), as demonstrated by indocyanine green angiography (ICGA) [1].

The pachychoroid disease spectrum comprises CSC, pachychoroid pigment epitheliopathy (PPE), pachychoroid neovasculopathy (PNV), peripapillary pachychoroid syndrome, and polypoidal choroidal vasculopathy (PCV) [2]. MPPE is not included within this classification, but it shares the same choroidal substrate and is best regarded as lying at the exudative extreme of the spectrum [1]. The defining features of the phenotype are pachyvessels, attenuation of the inner choroid and choriocapillaris, and choroidal vascular hyperpermeability, with accumulating evidence that choroidal venous congestion is the primary driver [2,3]. Eyes with this phenotype are therefore predisposed to choroidal vascular dysregulation and may be particularly susceptible to exudative decompensation under additional hemodynamic stress [2].

However, the exact pathophysiology remains incompletely understood. Acute intraocular pressure (IOP) reduction after glaucoma surgery lowers the tissue hydrostatic pressure within the choroid and thereby widens the transmural hydrostatic gradient across the choroidal vessel wall. This may promote fluid egress into the sub-RPE and subretinal spaces, and has been suggested to exacerbate CSC [4]. Consistent with this concept, serous retinal detachment accompanied by pachychoroid has been described after trabeculectomy, in which the exudative change was attributed to the same widening of the transmural gradient and consequent breakdown of the RPE barrier [5]. Postoperative corticosteroid use is also a recognized risk factor for CSC and pachychoroid-related exudation, and the two mechanisms may overlap clinically [2,6]. Herein, we report a case of MPPE developing after Ahmed® Glaucoma Valve (AGV) implantation.

## Case presentation

An 81-year-old man with exfoliation glaucoma (XFG) in the right eye was referred for uncontrolled IOP despite maximally tolerated medical therapy (tafluprost once daily and dorzolamide hydrochloride-timolol maleate twice daily). IOP was 48 mmHg, and BCVA was 20/20 in the right eye; in the left eye, BCVA was 20/16, and IOP was 16 mmHg. The right eye was pseudophakic, having undergone uncomplicated combined cataract surgery and trabeculotomy six years previously. His systemic history was notable for Parkinson's disease treated with levodopa, hypertension, and hyperlipidemia, and for a history of smoking, although details were unavailable. He had no diabetes mellitus and no history of systemic, inhaled, or periocular corticosteroid use, or of other medication associated with CSC.

Humphrey Field Analyzer 24-2 (Carl Zeiss Meditec AG, Jena, Germany) showed advanced glaucomatous damage, with a mean deviation of −22 dB; the vertical cup-to-disc ratio was 0.9, and the axial length was 24.03 mm. Fundoscopy revealed bilateral pachydrusen - large (>125 μm) drusenoid deposits with irregular outlines, scattered across the posterior pole, without PEDs or macular neovascularization. Preoperative optical coherence tomography (OCT) with enhanced depth imaging (SPECTRALIS®; Heidelberg Engineering, Heidelberg, Germany) demonstrated dilated pachyvessels in Haller's layer and attenuation of the inner choroidal layers, consistent with a pachychoroid phenotype; subfoveal choroidal thickness, measured manually from the outer border of the RPE to the chorioscleral interface, was 210 μm in the right eye (Table 1).

**Table 1:** Preoperative clinical characteristics The characteristics of the left eye are: BCVA 20/16; IOP 16 mmHg; pachydrusen without exudative change. BCVA, best-corrected visual acuity; HFA, Humphrey Field Analyzer; IOP, intraocular pressure; MD, mean deviation

Parameter	Right eye (affected)
Glaucoma diagnosis	Exfoliation glaucoma
Prior ocular surgery	Combined cataract surgery and trabeculotomy, 6 years earlier (uncomplicated)
Lens status	Pseudophakic
Systemic history	Parkinson's disease (levodopa); hypertension; hyperlipidemia; past smoking (no diabetes mellitus)
BCVA	20/20
IOP (mmHg)	48 (on maximal tolerated medical therapy)
Glaucoma severity (HFA 24-2 MD)	−22 dB
Vertical cup-to-disc ratio	0.9
Axial length (mm)	24.03
Drusen	Pachydrusen (large, >125 µm), bilateral
Subfoveal choroidal thickness (µm)	210
Choroidal morphology	Dilated pachyvessels in Haller's layer; inner choroidal attenuation

The patient underwent ciliary sulcus placement of an AGV (model FP7; New World Medical, Rancho Cucamonga, California, United States) tube in the superotemporal quadrant of the right eye to minimize corneal endothelial cell loss. The procedure was uneventful. IOP was 9 mmHg on postoperative day (POD) 1, reached a nadir of 5 mmHg on POD 7, and was 6 mmHg on POD 30. No hypotony-related complications were observed at any point, including hypotony maculopathy, ciliochoroidal effusion, shallow anterior chamber, choroidal folds, or choroidal detachment. Topical betamethasone 0.1% was administered four times daily from the day of surgery; the frequency was never increased, and the drop was discontinued on POD 93, after the SRD had resolved.

On POD 30, BCVA decreased to 20/100. Fundus photography revealed SRD extending from the macula to the inferior periphery, with scattered blot retinal hemorrhages in the right eye (Figure 1A); the retinal veins were of normal caliber and course, and the hemorrhages were considered to reflect his systemic hypertension. The left eye showed pachydrusen without exudative changes (Figure 1B). Compared with the preoperative OCT (Figure 1C, 1E, 1G), postoperative OCT demonstrated multiple PEDs at the macula, subretinal fluid extending inferiorly, and marked choroidal thickening with pachyvessel dilatation (Figure 1D, 1F, 1H); subfoveal choroidal thickness had increased from 210 to 362 μm.

**Figure 1:**
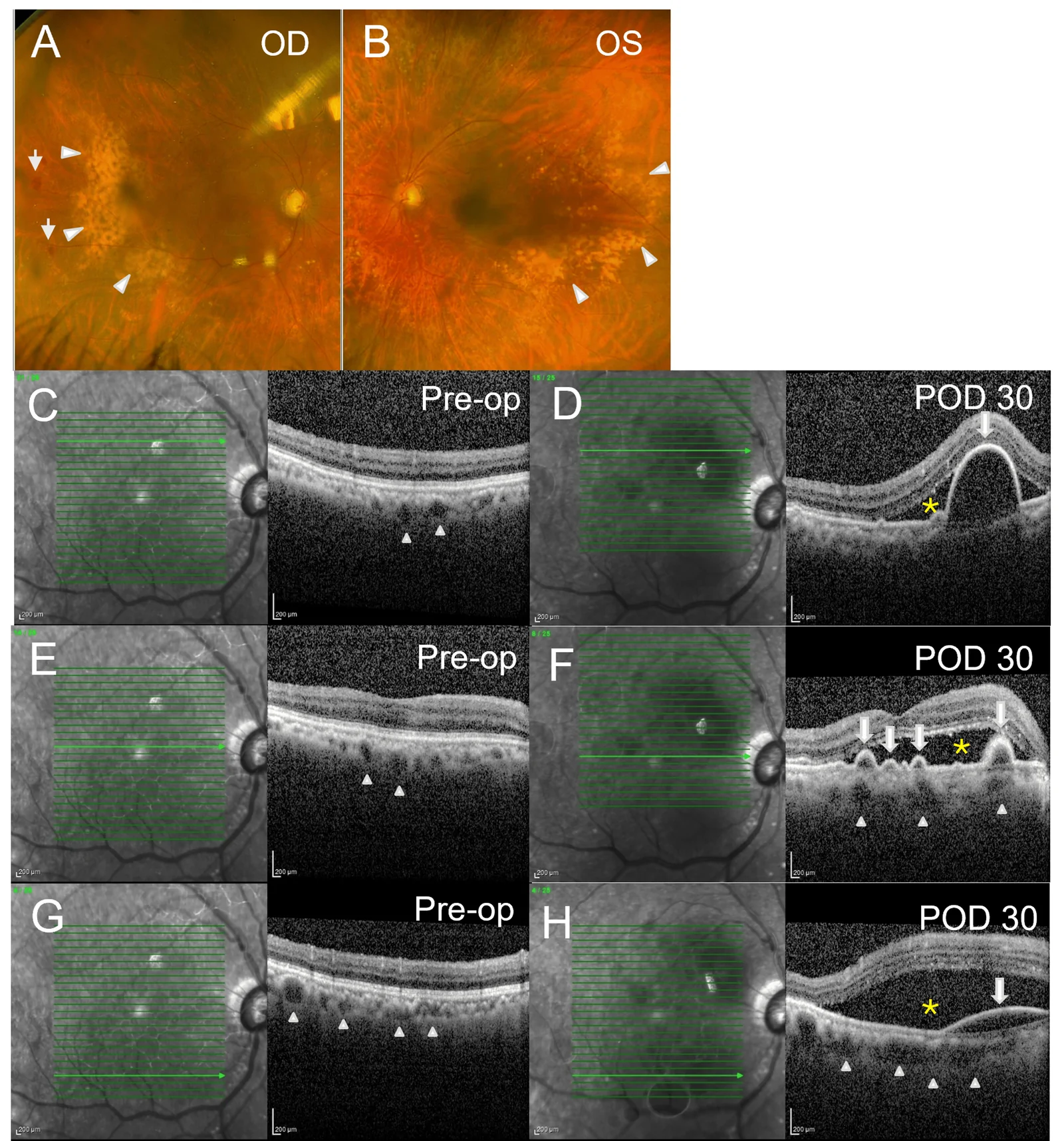
Fundus photographs and OCT findings at the onset of MPPE (POD 30). (A, B) Fundus photographs of the right and left eyes, respectively. Bilateral pachydrusen are visible on the temporal side of the posterior pole (arrowheads). In the right eye, optic nerve cupping, scattered blot retinal hemorrhages in the temporal peripheral retina (arrow), and serous retinal detachment extending from the macula to the inferior periphery are observed. (C–H) OCT cross-sections at the same location preoperatively (C, E, G) and at onset (D, F, H). Preoperative OCT (C, E, G) demonstrates dilated pachyvessels in Haller's layer with inner choroidal attenuation, consistent with pachychoroid features. Postoperative OCT (D, F, H) shows multiple pigment epithelial detachments at the macula (arrows) and serous retinal detachment extending to the inferior periphery (asterisks). Choroidal thickening with pachyvessel dilatation is evident at onset compared with preoperative findings (arrowheads). OCT, optical coherence tomography; MPPE, multifocal posterior pigment epitheliopathy; POD, postoperative day; Pre-op: preoperative

FA showed no early-phase leakage (Figure 2A). In the late phase, multifocal leakage was observed in the superior and inferior nasal macula, with additional leakage in the superior temporal region (Figure 2B). Mid- to late-phase ICGA revealed macular hyperfluorescence consistent with choroidal hyperpermeability in the right eye, with no corresponding hyperfluorescence in the left eye and no neovascular network or polypoidal lesion (Figure 2C, 2D). OCT angiography demonstrated multiple PEDs overlying dilated choroidal vessels, with no flow signal beneath the RPE on the outer retina-choriocapillaris slab to indicate macular neovascularization (Figure 2E, 2F). MPPE was diagnosed.

**Figure 2:**
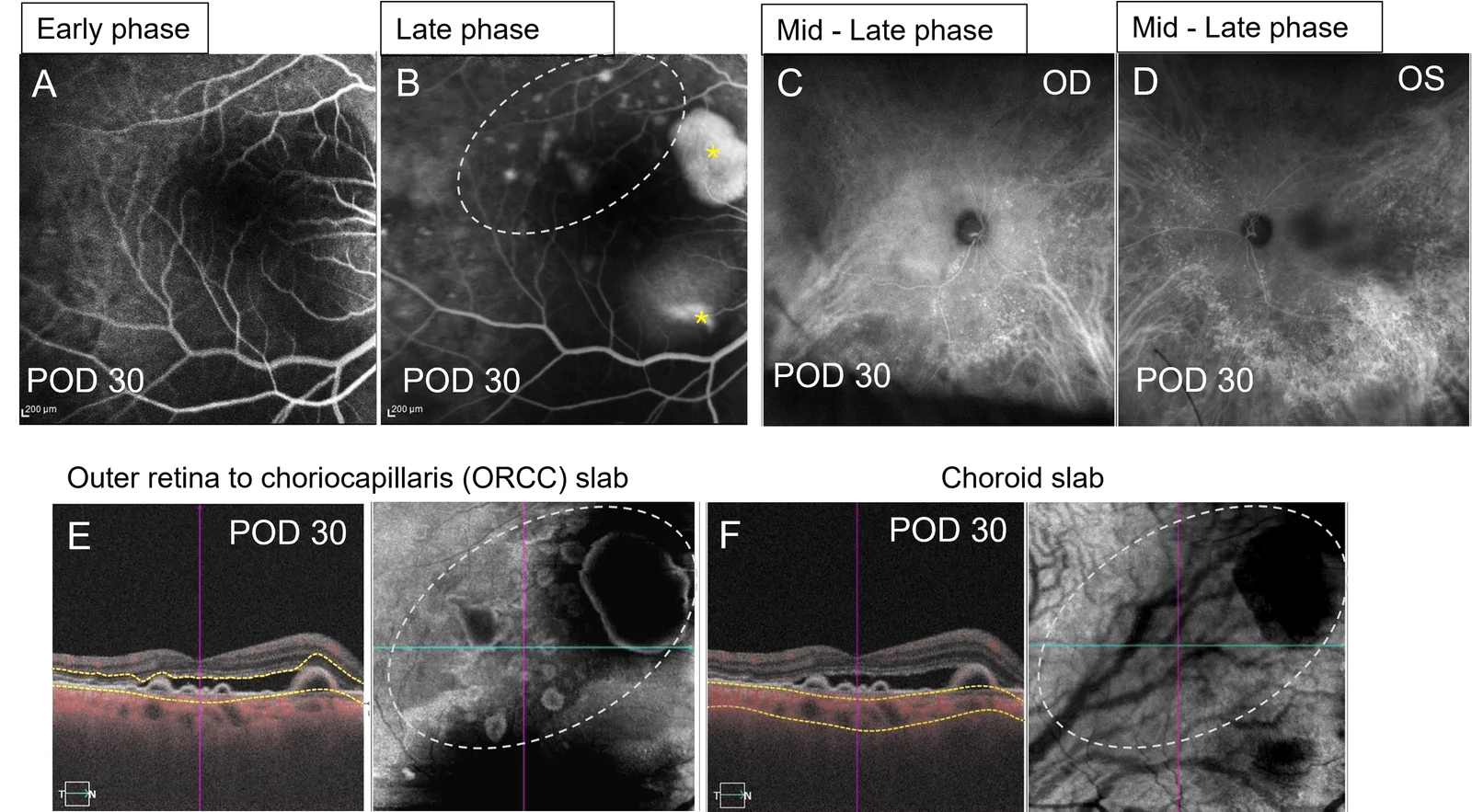
Multimodal angiography and OCT angiography findings at POD 30. (A, B) Fluorescein angiography (early phase, A; late phase, B). In the late phase, intense leakage is observed in the superior and inferior nasal macula (asterisks), with additional multifocal leakage in the superior temporal region (dotted outline). (C, D) Indocyanine green angiography of the right (C) and left (D) eyes. Mid- to late-phase macular hyperfluorescence in the right eye is consistent with choroidal hyperpermeability. No neovascular network or polypoidal lesion was evident in either eye. (E, F) OCT angiography (outer retina–choriocapillaris slab, E; choroid slab, F). Multiple pigment epithelial detachments are identified overlying dilated choroidal vessels, with no flow signal beneath the retinal pigment epithelium to indicate macular neovascularization. OCT, optical coherence tomography; POD, postoperative day

On POD 31, focal laser photocoagulation (577 nm yellow laser) was applied to the extrafoveal leakage points identified on FA (power 80-100 mW, spot size 100 μm, duration 100 ms, 25 spots). Two months after surgery, OCT demonstrated a marked reduction in PEDs and subretinal fluid compared with the baseline OCT findings at POD 30 (Figure 3A, 3B). Three months after surgery, the SRD had completely resolved (Figure 3C). Figure 3D and Table 2 summarize the postoperative course of IOP values and key clinical events.

**Figure 3:**
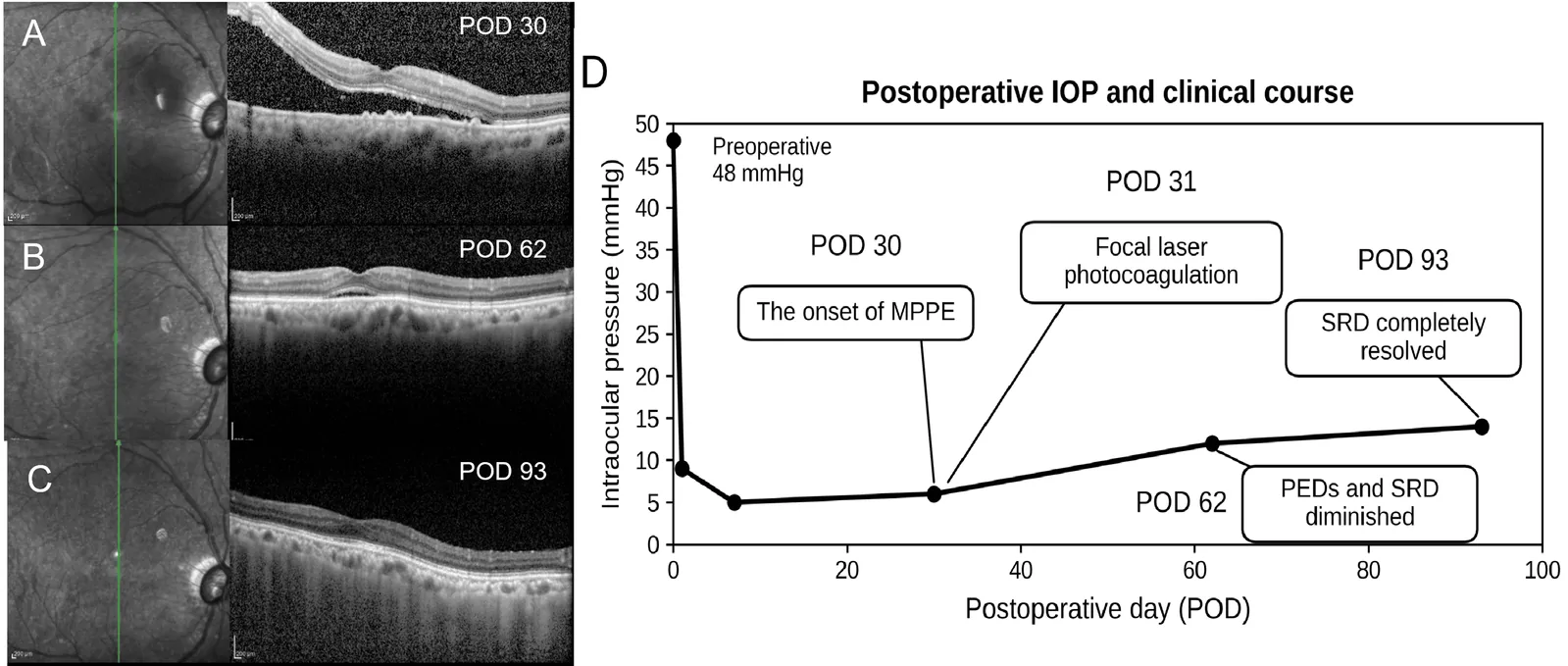
OCT findings and postoperative clinical course. (A–C) OCT at the macula on POD 30 (A), POD 62 (B), and POD 93 (C). The multiple pigment epithelial detachments and subretinal fluid present at onset (A) had markedly diminished by POD 62 (B), and the serous retinal detachment had resolved completely by POD 93 (C). (D) Time course of IOP with key clinical events. IOP fell from 48 mmHg preoperatively to a nadir of 5 mmHg on POD 7 and was 6 mmHg at the onset of MPPE on POD 30; the exudative changes regressed as IOP recovered to 12 mmHg on POD 62 and 14 mmHg on POD 93. OCT, optical coherence tomography; POD, postoperative day; MPPE, multifocal posterior pigment epitheliopathy; IOP, intraocular pressure

**Table 2:** Postoperative clinical course "—" indicates "not assessed". BCVA, best-corrected visual acuity; CT, choroidal thickness; FA, fluorescein angiography; IOP, intraocular pressure; MPPE, multifocal posterior pigment epitheliopathy; PED, pigment epithelial detachment; POD, postoperative day; SRD, serous retinal detachment

Time point	IOP (mmHg)	BCVA	Subfoveal CT (µm)	Clinical events
Preoperative	48	20/20	210	Baseline
POD 1	9	—	—	—
POD 7	5	—	—	No hypotony-related structural complication
POD 30	6	20/100	362	Onset of MPPE: multifocal PEDs and SRD
POD 31	—	—	—	Focal laser photocoagulation to extrafoveal FA leakage points
POD 62	12	—	—	PEDs and subretinal fluid diminished
POD 93	14	20/320	—	SRD completely resolved

Despite anatomical recovery, BCVA declined to 20/320 at the final visit, on POD 93. OCT at that visit showed disruption of the ellipsoid zone and external limiting membrane at the fovea.

## Discussion

Exudative choroidal and retinal changes following glaucoma surgery are rare, and the reported cases, variously described as CSC, serous retinal detachment, or hypotony maculopathy, are summarized in Table 3. They follow trabeculectomy [5,7,8], non-penetrating deep sclerectomy [9], and ab interno trabeculotomy [10]. Takakura et al. attributed CSC after trabeculectomy in a microphthalmic eye to venous congestion caused by scleral thickening [7]; our patient had a normal axial length and no known scleral abnormality, suggesting a different mechanism. One report concerns a glaucoma drainage device: Kokame et al. described serous retinal detachment with cystoid macular edema in hypotony maculopathy following Baerveldt implantation [11]. In nearly all of these reports, the exudation arose in the setting of frank hypotony or overt hypotony maculopathy.

**Table 3:** Reported cases of exudative choroidal and retinal pigment epithelial changes following glaucoma surgery BCVA, best-corrected visual acuity; CSC, central serous chorioretinopathy; FA, fluorescein angiography; IOP, intraocular pressure; MPPE, multifocal posterior pigment epitheliopathy; NS, not stated; PED, pigment epithelial detachment; POD, postoperative day; RPE, retinal pigment epithelium; SRD, serous retinal detachment; VEGF, vascular endothelial growth factor.

Study (Author, Year)	Age/Sex	Glaucoma Surgery (Indication)	IOP Course (mmHg)	Time from Surgery to Onset	Diagnosis	Key Choroidal/RPE Findings	Treatment and Outcome
Kokame et al., 2001 [11]	39 years/F	Pars plana vitrectomy with pars plana Baerveldt implant (uveitic glaucoma)	Hypotony (value not stated)	4 months	Hypotony maculopathy with SRD	Serous macular detachment with cystoid macular edema and choroidal folds	Resolved upon resolution of hypotony
Aydin et al., 2012 [8]	35 years/M	Trabeculectomy (traumatic glaucoma)	Hypotony from POD 2 (value not stated)	POD 2	Central serous retinal detachment	Choroidal leakage from traumatic perifoveal RPE defects	Observation; SRD resolved over 2 months
Takakura et al., 2014 [7]	46 years/M	Trabeculectomy (glaucoma in microphthalmos)	NS	NS	CSC	Serous macular detachment and multiple PEDs; smoke-stack leakage on FA; axial length 19.7 mm (microphthalmos)	NS; persistent vision loss
Shimokawa et al., 2020 [5]	66 years/F; 60 years/M	Trabeculectomy (neovascular glaucoma; 2 cases)	Hypotony after laser suture lysis (8 and 3)	POD 28 and POD 22	Hypotony maculopathy with SRD	SRD with pachychoroid; VEGF-independent exudation	Scleral re-suturing; IOP rose, and SRD improved in both
Barbosa et al., 2022 [9]	Late 70s/F	Non-penetrating deep sclerectomy (open-angle glaucoma)	2 (3 year postoperative)	3 years	Hypotony maculopathy	Macular folds with subretinal fluid	Topical steroid; resolved, recurred on withdrawal, stable on maintenance
Nakagawa and Ishii, 2025 [10]	71 years/F	Ab interno trabeculotomy (open-angle glaucoma)	4–6 postop	5 months	SRD	Increased choroidal thickness, vessel dilation, hyperpermeability, ciliochoroidal detachment	Observation; persisted 2 yr 7 mo, resolved after IOP spike
Present case, 2026	81 years/M	Ahmed Glaucoma Valve, FP7 (exfoliation glaucoma)	48 → 5 (POD 7); 6 at onset	POD 30	MPPE	Multifocal PEDs + SRD on a pachychoroid substrate; choroidal thickness 210 → 362 µm; no hypotony-related structural complication	FA-guided focal laser; SRD resolved by POD 93 as IOP recovered to 14 mmHg; BCVA 20/320

The present case differs in three respects. First, the exudation developed at POD 30 without any hypotony-related structural complication, so it cannot be attributed to hypotony maculopathy. Second, the pachychoroid substrate was demonstrable before surgery on morphological grounds: pachyvessels in Haller's layer with inner choroidal attenuation, despite an unremarkable subfoveal choroidal thickness of 210 μm, so the eye was predisposed rather than rendered susceptible by the surgery itself. Third, the phenotype was that of MPPE, multifocal PEDs with multifocal angiographic leakage and an inferior bullous exudative detachment, rather than a focal macular serous detachment. A PubMed search on September 7, 2026, using the terms (“multifocal posterior pigment epitheliopathy”) OR ((pachychoroid OR “central serous chorioretinopathy” OR “serous retinal detachment”) AND (“glaucoma surgery” OR trabeculectomy OR “glaucoma drainage device” OR Ahmed OR Baerveldt)), identified no previous report of MPPE following any glaucoma procedure.

The case fulfilled the features originally described for MPPE by Uyama et al., including multiple diffuse exudative lesions across the posterior pole, a flattened exudative detachment at the macula, and an inferior bullous retinal detachment [1]. Our patient showed multifocal PEDs and multiple discrete FA leakage points rather than a single focal leak, a shallow exudative detachment involving the fovea, and gravitational extension of subretinal fluid to the inferior periphery. Diffuse rather than focal RPE decompensation, together with the multifocal leakage and the marked choroidal hyperpermeability on ICGA, distinguished this presentation from typical CSC, in which one or a few focal leaks and a more circumscribed detachment are the rule [6]. Separating MPPE from a bullous variant of CSC is a matter of degree rather than of kind; we use the term MPPE because multifocality and diffuse posterior-pole involvement were the dominant features.

The preoperative OCT findings are consistent with an underlying pachychoroid phenotype. PPE, first described by Warrow et al., represents a non-exudative state in which RPE cells overlying dilated choroidal vessels are chronically exposed to elevated hydrostatic pressure from the expanded choroidal stroma [12]. MPPE may be conceptualized as a severe exudative manifestation of the same process. Notably, the preoperative subfoveal choroidal thickness of 210 μm lay within the range reported for healthy eyes (191-350 μm) and would not by itself support the diagnosis; the phenotype rested instead on the features now regarded as defining: pachyvessels, inner choroidal attenuation, and choroidal vascular hyperpermeability on ICGA [2,3]. What proved informative was not the baseline thickness but its change, from 210 to 362 μm. The drusen subtype was also informative. They were pachydrusen rather than soft drusen, a distinction that matters because pachydrusen are associated with increased Haller's layer thickness and attenuation of the overlying choriocapillaris and have been proposed to represent an epiphenomenon of the pachychoroid disease spectrum rather than a marker of age-related macular degeneration (AMD) [2]. Their presence therefore reinforces the pachychoroid interpretation and indicates that the RPE-Bruch's membrane complex had been under diffuse stress well before surgery, so that the eye likely entered surgery with an RPE less able to withstand an added hydrostatic load.

A mechanism related to AGV implantation may have contributed in this vulnerable eye. Trabeculectomy increases choroidal thickness and choroidal blood flow [13,14], and similar hemodynamic principles are likely to apply to AGV implantation. A low IOP represents a low tissue hydrostatic pressure within the choroid, which widens the transmural hydrostatic gradient across the choroidal vessel wall and promotes fluid movement into the choroidal stroma (Figure 4); Shimokawa et al. proposed this mechanism for serous retinal detachment with pachychoroid after trabeculectomy, albeit in eyes with frank hypotony maculopathy [5]. The reduction from 48 to 5 mmHg may thus have unmasked the latent exudative potential of the pre-existing pachychoroid substrate, and the parallel increase in choroidal thickness supports this. Consistent with such a susceptibility, all four risk factors for choroidal detachment after AGV implantation (exfoliation glaucoma, older age, pseudophakia, and systemic hypertension) were present [15]; although no choroidal detachment occurred, the shared background suggests a common vulnerability of the choroid to abrupt ocular decompression. The subsequent course is consistent with a bidirectional relationship: as IOP recovered to 12 mmHg at POD 62 and 14 mmHg at POD 93, the PEDs and subretinal fluid progressively diminished and the SRD resolved. Resolution therefore likely reflected not only focal laser photocoagulation but also normalization of IOP, which would have narrowed the transmural gradient; in previously reported cases, the serous detachment likewise resolved only after IOP rose, whether spontaneously or surgically [5,10,11].

**Figure 4:**
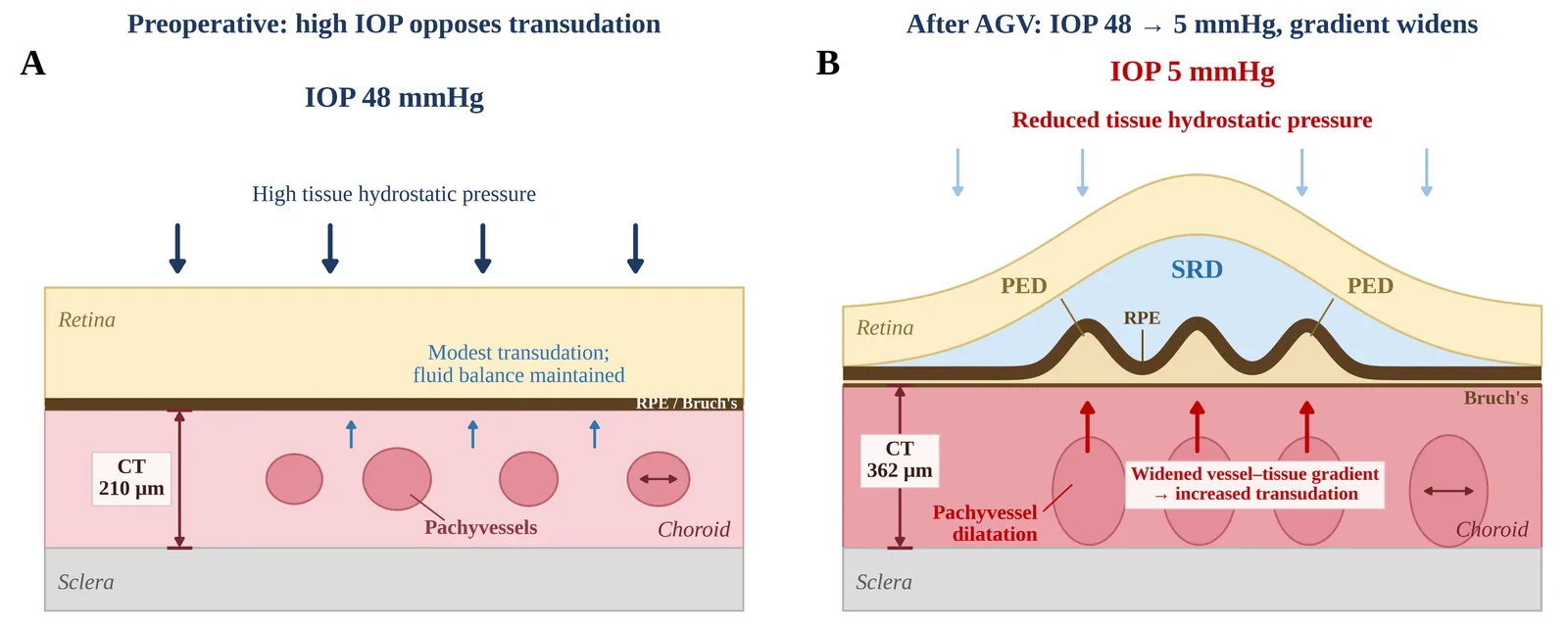
Proposed mechanism of exudative decompensation after acute IOP reduction, before (A) and after (B) Ahmed Glaucoma Valve implantation. IOP determines the tissue hydrostatic pressure within the choroid, which opposes transudation from the choroidal vessels. (A) Preoperatively, at an IOP of 48 mmHg, transudation is modest and fluid balance is maintained; subfoveal choroidal thickness is 210 μm. (B) After surgery, the fall in IOP to 5 mmHg reduces the tissue hydrostatic pressure and widens the transmural hydrostatic gradient across the choroidal vessel wall, increasing fluid movement into the choroidal stroma (solid red arrows), consistent with the mechanism proposed for pachychoroid-associated serous retinal detachment after trabeculectomy [5]. Fluid accumulates beneath the retinal pigment epithelium, elevating it from Bruch’s membrane to form PEDs, and passes into the subretinal space to produce an SRD. Subfoveal choroidal thickness rises to 362 μm. IOP, intraocular pressure; SRD, serous retinal detachment; PED, pigment epithelial detachment Image Credit: Authors; created using PowerPoint (Microsoft Corporation, Redmond, Washington, United States)

We also considered whether the AGV plate, sutured in the superotemporal quadrant, might have compressed the vortex vein ampulla or its scleral exit, but the evidence does not support this. Anatomically, the plate lies approximately 8-10 mm posterior to the limbus, whereas the vortex vein ampullae lie at or behind the equator. Functionally, vortex vein occlusion produced by scleral buckling causes sustained congestion in only a minority of eyes, because venovenous anastomoses connect the occluded sector to intact vortex veins within about three months [16]. The same compensation is observed experimentally: ligation of vortex veins in the monkey eye produces choroidal thickening and intervortex anastomoses within days, which largely resolve over the following weeks, and ligation alone did not produce serous retinal detachment [17]. Since exudation began at POD 30 and only one quadrant was instrumented, we do not regard plate-mediated compression as a plausible contributor.

Postoperative corticosteroid use is a known risk factor for CSC and pachychoroid-related exudation [2,6], and our patient was still receiving topical betamethasone four times daily at onset. Topical administration does not eliminate this concern, and a contributory role cannot be excluded. The temporal pattern, however, does not point to the steroid: it was begun immediately after surgery and never escalated, yet the exudative change appeared only at POD 30, in close temporal relation to the IOP nadir; and it was continued unchanged until POD 93 and stopped only after the SRD had resolved, so the exudation both developed and resolved during uninterrupted, constant-dose exposure. We therefore regard the hemodynamic changes induced by the IOP reduction, superimposed on the RPE fragility attributable to the pachychoroid phenotype, as the more probable precipitant, while recognizing that the two may have acted together.

Multimodal imaging excluded neovascular disease. OCT angiography demonstrated multiple PEDs overlying dilated choroidal vessels without macular neovascularization, and ICGA showed no neovascular network or polypoidal lesion, excluding neovascular AMD, PNV (defined as type 1 macular neovascularization arising over a pachychoroid) [18], and PCV; the pachydrusen phenotype further argues against a drusen-driven AMD background. Hypotony maculopathy was excluded, as no chorioretinal folds, ciliochoroidal effusion, shallow anterior chamber, or choroidal detachment were observed at any point. Hypertensive chorioretinopathy was also considered, given the hypertension and scattered blot hemorrhages; however, no cotton-wool spots, arteriolar narrowing, or optic disc swelling were present, blood pressure was well controlled, and the fellow eye showed no exudative change despite the same systemic exposure. The absence of neovascularization is consistent with PED formation driven by hydrostatic and hemodynamic rather than neovascular factors, supporting classification at the exudative end of the pachychoroid disease spectrum.

The choice of treatment deserves comment, since no guideline addresses this situation. Half-dose PDT is regarded as the treatment of choice for chronic CSC, with observation preferred initially in acute CSC, although exceptions based on patient-specific characteristics are explicitly allowed [6]; neither recommendation is derived from eyes such as ours, in which a bullous exudative detachment arose acutely on a pachychoroid substrate after an abrupt fall in IOP. We elected to treat rather than observe because the detachment involved the fovea and extended to the inferior periphery and BCVA had already fallen to 20/100. Focal laser was preferred to PDT because the leakage points were discrete and extrafoveal and therefore amenable to direct treatment, and because FA-guided focal laser photocoagulation has recently been reported to resolve the exudative changes of MPPE, albeit with additional treatment for newly developing leakage points [19]. The subsequent course, however, does not allow the contribution of the laser to be separated from that of the recovering IOP. Anatomical resolution was not matched by visual recovery: BCVA declined to 20/320 by POD 93 despite complete resolution of the SRD. Disruption of the ellipsoid zone and external limiting membrane indicates that the photoreceptor-RPE interface sustained structural damage during the period of foveal detachment, and the advanced glaucomatous optic neuropathy present before surgery (mean deviation −22 dB) is likely to have limited functional recovery. The laser burns were confined to extrafoveal locations and are unlikely to account for the central visual loss.

This report has several limitations. Scleral thickness was not measured; since scleral thickening is associated with impaired vortex vein outflow and with the pachychoroid phenotype in CSC, its contribution can neither be established nor excluded [20]. Wide-field ICGA was not performed, so vortex vein congestion and intervortex anastomoses could not be assessed [3]. The patient was receiving topical corticosteroid at onset, as discussed above. Follow-up ended at POD 93, when the SRD had just resolved, and the corticosteroid drop was discontinued; the BCVA of 20/320 recorded then may therefore not represent the final functional outcome, and longer observation would be required to determine whether the outer retinal changes and visual acuity recover further. Finally, causality cannot be established from a single case: the associations described here are suggestive rather than confirmatory.

## Conclusions

This was a rare case of MPPE developing after AGV implantation in an eye with a pachychoroid phenotype. A marked, rapid IOP reduction may contribute to exudative decompensation in a susceptible choroid, even in the absence of hypotony-related structural complications and despite an unremarkable preoperative choroidal thickness. Choroidal morphology should be assessed before glaucoma surgery in such eyes, with careful postoperative retinal monitoring.
